# Fucoidan’s Molecular Targets: A Comprehensive Review of Its Unique and Multiple Targets Accounting for Promising Bioactivities Supported by In Silico Studies

**DOI:** 10.3390/md22010029

**Published:** 2023-12-30

**Authors:** Ahmed Zayed, Dalal A. Al-Saedi, Emmanuel Ofosu Mensah, Osman Nabayire Kanwugu, Parise Adadi, Roland Ulber

**Affiliations:** 1 Institute of Bioprocess Engineering, Rheinland-Pfälzische Technische Universität Kaiserslautern-Landau, Gottlieb-Daimler-Straße 49, 67663 Kaiserslautern, Germany; 2 Department of Pharmacognosy, College of Pharmacy, Tanta University, El-Guish Street (Medical Campus), Tanta 31527, Egypt; 3 Department of Biochemistry, Faculty of Sciences, King Abdulaziz University, Jeddah 21589, Saudi Arabia; dalal.alsaadi@hotmail.com; 4 Faculty of Ecotechnology, ITMO University, Lomonosova Street 9, Saint Petersburg 191002, Russia; emensakh@itmo.ru; 5 Institute of Chemical Engineering, Ural Federal University, Mira Street 28, Yekaterinburg 620002, Russia; nabayire@gmail.com; 6 ARC Centre of Excellence in Synthetic Biology, School of Natural Sciences, Macquarie University, Sydney, NSW 2109, Australia; 7 Department of Food Science, University of Otago, Dunedin 9054, New Zealand; pariseadadi@gmail.com

**Keywords:** bioactivities, fucoidan, inflammation markers, molecular mechanisms, signaling pathways

## Abstract

Fucoidan is a class of multifunctional polysaccharides derived from marine organisms. Its unique and diversified physicochemical and chemical properties have qualified them for potential and promising pharmacological uses in human diseases, including inflammation, tumors, immunity disorders, kidney diseases, and diabetes. Physicochemical and chemical properties are the main contributors to these bioactivities. The previous literature has attributed such activities to its ability to target key enzymes and receptors involved in potential disease pathways, either directly or indirectly, where the anionic sulfate ester groups are mainly involved in these interactions. These findings also confirm the advantageous pharmacological uses of sulfated versus non-sulfated polysaccharides. The current review shall highlight the molecular targets of fucoidans, especially enzymes, and the subsequent responses via either the upregulation or downregulation of mediators’ expression in various tissue abnormalities. In addition, in silico studies will be applied to support the previous findings and show the significant contributors. The current review may help in understanding the molecular mechanisms of fucoidan. Also, the findings of this review may be utilized in the design of specific oligomers inspired by fucoidan with the purpose of treating life-threatening human diseases effectively.

## 1. Introduction

Recent developments in the pharmaceutical industry have seen the exploitation of various natural products, such as polysaccharides [1,2]. These polysaccharides are integral to organisms such as plants, animals, seaweed, and microorganisms that confer structural integrity and serve other roles [1]. Research has indicated these polysaccharides to possess biological activities such as antioxidant, anticancer, anti-inflammatory, antidiabetic, antiaging, and cardioprotective activities [3,4,5]. Generally, polysaccharides have attracted great attention in the medicinal and pharmaceutical industries because of their high activity, biodegradability, biocompatibility, low toxicity, and hydrophilic nature [6]. Commonly known polysaccharides are ulvan, levan, chitosan, agar, xanthan, *β*-glucan, xylan, laminaran, pectin, and fucoidan, among others [1,7].

Particularly, fucoidan is a unique polysaccharide characterized by the presence of sulfate esters attached to specific carbon groups on the main *α*-L-fucopyranose chain [8]. This group of polysaccharides is predominant in the cell walls of brown seaweeds, especially those of the genus *Fucus* and others. Research has shown different sources of fucoidan, such as *Fucus vesiculosus*, *Ascophyllum nodosum*, *Laminaria japonica*, *Sargassum latissimi*, and *Ecklonia cava* [9,10]. They are structurally complex polysaccharides made up of different monomers such as fucose (main sugar monomer), galactose, mannose, xylose, and uronic acids [11]. Additionally, research has shown fucoidan to possess residues of proteins, minerals, and other phenolic compounds [9,12]. The skeletal structure of fucoidans is made up of either repeating units of (1→3)– or alternating (1→3)– and (1→4)– linked *α*-fucopyranose units. Fucoidans are negatively charged due to the presence of their sulfate esters and the carboxylic moieties found in other species [13].

As part of their rich composition and structural heterogeneity, research has also proven fucoidan to possess different biological activities such as anticancer [14], antiaging [15], antioxidant [16], anti-inflammatory [17], anticoagulant [18], antimicrobial [19], antiatherogenic [20,21], gastroprotective [22], and cardioprotective activities [9]. General reports have indicated that the biological activities of fucoidans are dependent on certain factors, such as the molecular weight, the extraction method, the degree of sulphation, the source of fucoidan, and the type of sugar monomers [11]. The evidence of these activities has led to growing interest in the use of fucoidans in various industries [9]. Among them, fucoidan has been used in the pharmaceutical industry in the development of drug delivery systems such as nanoparticles, liposomes, micelles, and semi-solid formulations, among others [23]. Research has shown fucoidans to possess certain properties that make them ideal for the development of delivery systems, such as controlled, biodegradable, biocompatible, low-toxicity, available, cost-effective, safe, and stable delivery with increased bioavailability [24]. Additionally, another component of fucoidan, which plays a key role in the pharmaceutical industry, is its hydrophilic nature. Fucoidan is highly soluble in aqueous media, which is very important in drug delivery [24]; however, there are certain discrepancies that have been associated with this.

**Figure 1:**
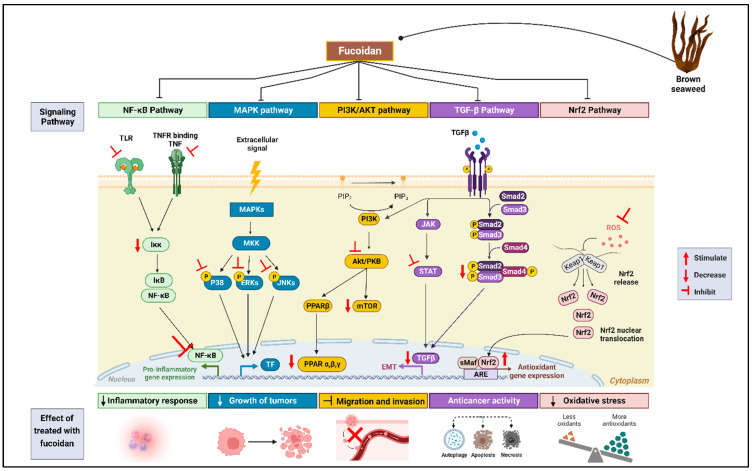
A schematic illustrating the various signaling pathways of fucoidan in relation to different bioactivities (Created with BioRender, Agreement number: KU263BTHW3).

Like all polysaccharides, fucoidan cannot freely pass through the cytomembrane; as such, it needs to bind certain receptors, such as pattern-recognition receptors (PRR). Research has indicated that several polysaccharides, including fucoidan, bind to receptors such as Toll-like receptors (TLRs), scavenger receptors (SRs), and C-type lectin receptors (CLRs) [6,25]. Therefore, this review seeks to describe several pharmaceutical applications of fucoidan and its molecular targets, supported by in silico studies. The multiple biological activities of fucoidan are mediated by its interaction with various proteins and signaling pathways, including NF-κB, MAPKs, TLRs, transforming growth factor Beta (TGF-β), nuclear factor erythroid 2-related factor 2 (Nrf2), and others, as shown in Figure 1. The effects of fucoidan on various molecular targets are discussed below and summarized in the table.

## 2. Search Strategy

Various databases, including SCOPUS, Web of Science, PubMed, Google Scholar, and the Scientific Electronic Online Library (SciELO), were searched for published research articles on different biological activities of fucoidan and their various interactions. The search phrases used for this review included: structure of fucoidan, molecular targets of fucoidan, biological activities of fucoidan, interaction of fucoidan with signaling pathways such as NF-κB, MAPK, PI3K/AkT, TGF-β, TNF-α, Nrf2, and VEGF, effect of structural properties of fucoidan on signaling pathways, impact of fucoidan dose on signaling pathways, interaction of fucoidan with receptors (including TLRs and EGFRs), how does fucoidan elicit biological activities by targeting these receptors, interaction of fucoidan with enzymes, stimulation of enzymes by fucoidan, toxicity of fucoidan, dose of fucoidan that induces toxicity, and in vivo toxicological studies of fucoidan. Priority was given to research articles within a period of 10 years, from 2013 to 2023; however, in certain cases, an exception was made.

## 3. Potential Molecular Targets of Fucoidan

### 3.1. Interaction with Signaling Pathways

#### 3.1.1. Nuclear Factor Kappa B (NF-κB) Pathway

NF-κB is an inducible transcription factor that regulates the expression of several genes linked to inflammation (a part of the body’s immune response) and cell survival. However, the aberrant activation of NF-κB has been linked to chronic inflammation as well as the induction of tumors and the survival of cancer cells. Under a normal physiologic state, NF-κB is sequestered as an inactive complex in the cytoplasm by inhibitory proteins known as inhibitors of κB (IκBs). Under perturbed conditions, IκBs are phosphorylated then targeted for degradation; thus, NF-κB becomes available to translocate into the nucleus to activate its target genes [26]. Fucoidan exhibits its anti-inflammatory activity partly by inhibiting the NF-κB-dependent expression of proinflammatory cytokines, as shown in Figure 1. In this regard, Sanjeewa et al. have reported that fucoidan promotes the stabilization of IκB-α by inhibiting its phosphorylation in a zebrafish model. The translocation of NF-κB was suppressed, thus decreasing the production of nitric oxide (NO). This study further demonstrated the effects of fucoidans against inflammation [27]. Similar observations were made in BV2 microglia cells [28]. Also, as a regulator, NF-κB tends to mediate inflammatory response by stimulating the release of cytokines such as TNF-α. Fucoidan reportedly inhibited the phosphorylation of NF-κB and subsequently downregulated the effects of the pro-inflammatory cytokines α/IFN-γ, IL-1β, and IL-6 [29]. Elsewhere, fucoidan decreased the phosphorylation of NF-κB, which in turn downregulated the mRNA expression of TNF-α tumor necrosis factor (TNF-α), reducing the effect of ophthalmic inflammation in ARPE-19 cells [30]. Jeong et al. indicated that fucoidan also had a role in the inhibition of the nuclear translocation of NF-κB, which led to the downregulation of inducible nitric oxide synthase (iNOS) and cyclooxygenase-2 (COX-2) in RAW 264.7 macrophages [31]. Others have demonstrated the anti-inflammatory activity of fucoidan via the NF-κB pathway and found that fucoidan (IC_50_ = 4.3 µg/mL) inhibited COX-2 with a higher selective index (Ig IC_80_ COX-2/COX-1–1.55) compared to the control drug, indomethacin (Ig IC_80_ COX-2/COX-1–0.09). Apparently, fucoidan exhibited a higher binding affinity to COX-2′s active site than the synthetic drug due to the presence of polyphenols, fucose, and sulfate [32]. Furthermore, phosphorylation on IKKα and IKKβ was decreased after incubation with fucoidan, thus attenuating the inflammatory response. A subsequent in vivo zebrafish model confirmed the anti-inflammatory activity of fucoidan, where iNOS and COX-2 were inhibited, thus decreasing NO production [33], as shown in Figure 1.

There have been reports suggesting that fucoidan directly interferes with the DNA binding activity of NF-κB. For instance, Shu et al. reported that, in addition to inhibiting the nuclear translocation of the p65 subunit of NF-κB, fucoidan treatment suppressed the DNA binding activity of NF-κB rheumatoid arthritis fibroblast-like synoviocytes [34]. Similarly, fucoidan regulated the progression of pancreatic cancer through the upregulation of cytoplasmic 1κB levels with a concomitant inhibition of NF-κB [35]. Lee et al. likewise showed that fucoidan ameliorated NF-κB activation by preventing the translocation of p65-NF-κB in human cancer cells and inhibiting the degradation of IκB [36]. Fucoidan further inhibited the expression of M2-type chemokine (CCL22) and tumor cell migration via suppressing p65-NF-κB phosphorylation and nuclear translocation in the human hepatoma cell line [37]. The previous literature has not reported on the exact relationships between fucoidan and NF-κB phosphorylation or the mechanism involved. However, several reports indicated that the high content of sulfate and fucose groups may be responsible for this activity, but it was not mechanistically proven [38]. Additionally, other studies have indicated the targeting of the active site of IκB—kinase beta (mainly two serine residues, Ser 177 and Ser 181) for the possible inhibition of phosphorylation [39]. As such, for the prospective mechanism involved, the sulfate ester groups or hydroxy-methyl groups of fucoidan may be suggestive of how they react with these Ser residues to prevent dephosphorylation.

On the contrary, the activation of NF-κB is important in improving immunogenicity and immunity. In this regard, NF-κB acts in a cascade of reactions, first as a regulator for the differentiation and maturation of B cell as well as the formation of lymphoid tissue. Furthermore, NF-κB plays a pivotal role in the survival of B-cells as well as lymphoid regeneration [40]. Additionally, NF-κB contributes to the development, activation, differentiation, and survival of T-cells [41]. Tarbasa et al. reported the activation of the NF-κB pathway in RAW264.7 murine macrophage cells and natural killer (NK-92) cells after incubation with fucoidan for 24 h [42]. Also, fucoidan induced T cell development and maturation by forming a TCR/CD3 complex on the cell surface, which in turn allowed for the nuclear translocation of NF-κB to fully activate T cells [43].

#### 3.1.2. Mitogen-Activated Protein Kinase (MAPK) Pathway

MAPK signaling pathways are key regulators of eukaryotic transcriptional responses, mainly involved in relaying, amplifying, and integrating signals from a diverse range of stimuli and eliciting an extracellular signal [44]. They are characterized by the presence of proline-directed serine/threonine protein kinases and distantly related to cyclin-dependent kinases. An MAPK pathway is made up of mainly three signaling pathways, including extracellular signal-regulated kinases 1 and 2 (ERK1/2), c-Jun N-terminal kinase (JNK), and p38 signaling families [45,46]. They are widely known to play a role in cell proliferation, embryonic development, and apoptosis [47]. The ERK pathway plays a pivotal role in cell proliferation and is generally phosphorylated and activated by cell surface receptors such as receptor tyrosine kinases, receptor serine kinases, cytokine receptors, integrins, and G-protein-coupled receptors in response to growth factors. On the other hand, JNK pathway kinases are kinases that are phosphorylated and activated in response to cellular stress, including UV radiation, heat shock, ionizing radiation, and oxidative stress, among others [45]. With regard to the p38 signaling pathway, it is activated under both environmental and cellular stresses, including hypoxia, ischemia, and inflammation [48]. Different studies have highlighted the regulation of the MAPK signaling pathway by fucoidan to prevent different diseases.

In DU-145 prostate cancer cells, treatment with fucoidan (1000 µg/mL) for 24 h reduced the expression of phosphorylated ERK and p38 (Figure 1), thus decreasing the growth of tumors in the cells [49]. Boo et al. demonstrated that fucoidan induces apoptosis in prostate cancer cells via the activation of ERK1/2 and the downregulation of p38 [50]. Similarly, fucoidan treatment (400 µg/mL) inhibited ERK phosphorylation in hepatocellular carcinoma cells. Interestingly, the treatment with fucoidan augmented the phosphorylation of p38 MAPK. The phosphorylation of p38 MAPK reduced the expression of Bcl2 proteins and controlled the translocation of Bax, whereas the inhibition of the ERK pathway activated Bax, resulting in the induction of apoptosis [51]. A similar effect was observed in mice breast cancer models after the administration of fucoidan [52]. Park et al. also revealed that fucoidan exerted its anti-inflammatory activity against brain macrophage cells by inhibiting the phosphorylation of the ERK, JNK, and p38 pathways in a dose-dependent manner [28]. Furthermore, fucoidan significantly inhibited adipogenesis in 3T3-L1 preadipocytes by downregulating both mRNA and the protein expression of p38 MAPKα and p38 MAPKβ. This effect subsequently led to the inhibition of ERK and JNK phosphorylation, which in turn decreased the expression of peroxisome proliferator-activated receptor gamma (PPARγ), i.e., a regulator of adipocyte differentiation [53]. The administration of fucoidan also inhibited the production of nitric oxide during inflammation by downregulating the phosphorylation of ERK and p38 [54].

Contrary to the above, fucoidan has also been reported to activate MAPKs in NK cells [42]. Additionally, a series of inflammations induced by lipopolysaccharides (LPSs) were reduced upon treatment with fucoidan via the inhibition of MAPK-mediated gene transcription [38]. Fucoidan increased immunity against visceral leishmaniasis partly by activating the p38 MAPK and ERK1/2 pathways. In terms of its mechanism, a decrease in p38 caused a reduction in IL-12, whereas the deactivation of ERK1/2 suppressed the development of TNF-α involved in inflammation [55]. Also, the molecular mechanism of how fucoidan stimulates nitric oxide (NO) production and inducible nitric oxide synthase (iNOS) activation via p38 MAPK inhibitors (i.e., SB203580 and PD98059) has been reported. Generally, SB203580 competes with other compounds like fucoidan for the active site on p38 MAPK to inhibit phosphorylation and suppress the production of NO as well as iNOS activation. However, SB203580 activation had no inhibitory effects on the fucoidan-induced phosphorylation of p38 MAPK [56]. Sapharikas et al. demonstrated that fucoidan enhances monocyte recruitment via the activation of the ERK and p38 MAPK pathways. The fucoidan-induced effect on the monocytes was abrogated by the ERK inhibitor PD98059 and the p38 inhibitor SB203580 [57].

#### 3.1.3. PI3K/AKT Pathway

Phosphatidylinositol 3-kinase/protein kinase B (PI3K/AKT) is an important signal transduction system which connects oncogenes and many receptor classes to essential cellular functions, including cell proliferation, survival, growth, and metabolism [58]. PI3K belongs to a group of lipid kinases that phosphorylate the 3-hydroxyl of the inositol ring of phosphatidylinositol lipids in the plasma membrane [59]. They are classified into three categories (Class I, II, and III) based on their different structures and lipid substrate preferences [60]. Among these categories, class I PI3K has been well studied and understood because of its link to the development of cancer [58]. PI3K is generally activated by growth factors (including epidermal growth factors (EGF), platelet-derived growth factors (PDGF), and/or insulin-like growth factors), cytokines, and hormones. Additionally, small GTPases, such as Ras and RAB5, tend to activate PI3K [58,61]. On the other hand, AKT is a serine/threonine kinase which functions as an effector of PI3K. Upon PI3K activation, AKT is translocated through its PH domain to the inner membrane, where it is further phosphorylated (at Thr 308) by PDK1 [62]. This phosphorylated AKT subsequently translocates from the cell membrane to different parts of the cell to perform its main functions through phosphorylating various downstream substrates [63]. These pathways therefore work together and have been linked to different forms of cancer [64] and diseases such as diabetes [65] and inflammation [66], among others.

The effect of fucoidan on the PI3K/AKT pathway has been reported in various studies. The anticancer activity of fucoidan in DU-145 PC cells has been reported to be mediated by the fucoidan-induced inhibition of PI3K/AKT phosphorylation [49]. Liu et al. similarly demonstrated that fucoidan inhibited the gene transcription and protein expression of PI3K while at the same time suppressing the phosphorylation of AKT in ovarian cancer cells, as shown in Figure 1. The downregulation of the PI3K/AKT pathway by fucoidan contributed, in part, to a reduced expression of CDK-4, CDK-6, cyclin-E, and cyclin-D1, consequently halting cancer cell growth while inducing the apoptosis of the cancer cells [67]. In another study, fucoidan inhibited the PI3K/AKT pathway both in vitro and in vivo, which contributed to the inhibition of cancer cell proliferation [68].

The mechanistic target of the rapamycin (mTOR) signaling pathway is complexly interwind with the PI3K/AKT pathway and plays a role in cell growth and survival and as a target in cancer [69]. Fucoidan has been found to inhibit the AKT-mediated activation of mTOR, leading to the suppression of mTOR signaling, subsequently affecting cell growth and proliferation [70]. Deng et al. likewise showed that fucoidan inhibited the phosphorylation of the PI3K/AKT pathway, which in turn suppressed mTOR, thus slowing the development of metabolic syndrome. Additionally, the downregulation of the PI3K/AKT-mTOR pathway was associated with the reduced expression of SREBP-1c and PPARγ in the liver, thereby preventing the risk of cardiovascular diseases [70,71]. Similarly, fucoidan suppressed the phosphorylation of the PI3K/AKT pathway, which further downregulated the phosphorylation of the mTOR signaling pathway in HT-29 colon cancer cells, consequently leading to the suppression of the migration, invasion, and proliferation of cancer cells [72,73]. Elsewhere, a treatment with fucoidan inhibited the expression of PI3K and AKT. The downregulation of the PI3K/AKT pathway, in turn, resulted in decreased phosphorylation of mTOR (including the targets 4E-BP1 and p70S6K). The inhibition of these pathways suppressed the growth of tumor cells [36]. Chen et al. reported that fucoidan induced apoptosis in A549 and H1650 cells after 48 h of incubation by downregulating the expression of the mTOR signaling pathway and its downstream proteins, p-S6K, p-P70S6K, and p-4EBP1 [70].

In contrast to the above studies, fucoidan treatment enhanced neuron protection by activating the PI3K/AKT pathway to prevent apoptosis induced by MPP^+^ in SH-SY5Y cells [74]. A similar observation was reported by Wang et al., who reported that fucoidan protected neurons from apoptosis by activating the PI3K/AKT pathway via enhanced phosphorylation [75].

#### 3.1.4. Transforming Growth Factor-Beta (TGF-β) Pathway

TGF-β is a multifunctional cytokine that plays a major role in several cellular mechanisms and physiological processes, including cell growth, differentiation, death, and migration [76]. The binding of TGF-β to respective receptors activates the signal transduction of Smad via phosphorylation, thus forming a series of Smad complexes, which are then translocated into the nucleus to mediate the transcription of target genes [77]. Studies have shown the role of TGF-β in the onset of various diseases, including cancer, hypertension, autoimmune disease, fibrosis, osteoporosis, and inflammatory disorders [78]. TGF-β particularly plays a dual role in the etiology and pathogenesis of cancer. While in the early stages, TGF-β suppresses the proliferation of tumor cells, TGF-β also promotes the aggressiveness and metastasis of advanced tumors [79].

Fucoidan has been reported to interact with TGF-β in response to disease pathogenesis. Li et al. [80] reported that levels of TGF-β were upregulated in hepatic sinus endothelial cells and inflammatory cells during liver fibrosis. The activation of TGF-β further upregulates Smad via phosphorylation in the nuclear region, inducing liver necrosis and autophagy. However, fucoidan treatment suppressed the growth of tumors by downregulating TGF-β, as shown in Figure 1. In addition, the phosphorylation of Smad significantly inhibited this growth [80]. Similarly, fucoidan attenuated radiation-induced fibrosis by inhibiting the TGF-β pathway. The mRNA expression of the Smad 3 and Smad 4 complexes was also reduced following fucoidan treatment, leading to the suppression of collagen 1 accumulation [81]. Also, fucoidan decreased the level of TGF-β receptors, i.e., TGF-βRI and TGF-βRII in MDA-MB-231 and MCF-7 human breast cancer cells, by enhancing the proteosome-mediated ubiquitination of such receptors. This, accordingly, affected the phosphorylation of the Smad 2 and 3 complexes as well as the expression of the Smad 4 complex [82]. The anticancer activity of fucoidan in gastric cancer was also shown to be mediated by a reduction in TGF-β secretion [83].

The activation of TGF-β induced an epithelial–mesenchymal transition (EMT) of the retinal pigment epithelium (RPE), a key process in the pathogenesis of proliferative vitreoretinopathy. However, the treatment of RPE cells with fucoidan reversed this effect and hence protected the retina from detachment. Fucoidan treatment decreased the phosphorylation of Smad 2 and 3, which was accompanied by the downregulation of α-smooth muscle actin (α-SMA) and fibronectin [84]. A similar effect was observed by Wang et al., where fucoidan exhibited anti-EMT activity against pulmonary fibrosis. The authors of this study reported that fucoidan suppressed TGF-β-induced EMTs through the ERK signaling pathway [85]. Also, in diabetic nephropathy models, fucoidan inhibited the TGF-β pathway, which resulted in a reduced accumulation of extracellular matrix proteins, including α-SMA and connective tissue growth factor. In addition to the decreased phosphorylation of Smads (Figure 1), the fucoidan treatment inhibited the phosphorylation and activation of AKT, ERK, and p38 [86]. Hsu et al. likewise demonstrated that fucoidan inhibited irradiation-induced fibrosis by downregulating the TGF-β pathway [87].

#### 3.1.5. Nuclear Factor Erythroid 2-Related Factor 2 (Nrf2) Pathway

Nrf2 is a basic leucine zipper transcription factor made up of a cap ‘n’ collar and encoded by the gene NFE2L2. Basically, it is composed of seven Nrf2-ECH homology (Neh) domains, ranging from Neh1 to Neh7. Regions including Neh1, 3, and 6 are located in the C-terminal domain of Nrf2, where Neh1 harbors the CNC/bZIP region, which allows for dimerization with small musculoaponeurotic fibrosarcoma (Maf) proteins in the nucleus, facilitating the binding of Nrf2 with DNA [88,89]. The N-terminal domain of Nrf2 is dominated by Neh2, which allows for the binding of Nrf2 to its cytosolic Kelch-like ECH-associating protein (Keap1). Inactive Nrf2 is bound to Keap1 through the DLG and ETGE motifs present in the Neh2 domain. The binding of Nrf2 to Keap1 increases its possibility for proteosome degradation. Following a thiol modification of cysteine residues in Keap1 after oxidative stress or contact with activators, Nrf2 dissociates from Keap1 and becomes active [90]. After activation, Nrf2, through the antioxidant-response element (ARE), upregulates the expression of antioxidant enzymes such as heme oxygenase 1 (HO-1), catalase (CAT), superoxide dismutase (SOD), and glutathione peroxidase (GPx). These antioxidant enzymes and molecules in turn mitigate the detrimental effects of oxidative stress on nucleic acids, proteins, and/or plasma lipids. Additionally, Nrf2 is coupled to the gene responsible for this; hence, its upregulation and downregulation have been linked to the pathogenesis of certain diseases, such as cancer, hypertension, diabetes, Alzheimer’s, cataract, and others [89,91].

Previous studies have revealed that fucoidan interacts with the Nrf2 pathway in relation to exhibiting some of its biological activities. For instance, the treatment of human hepatocyte HL-7702 cells with fucoidan ameliorated the effects of acetaminophen-induced hepatotoxicity by activating the Nrf2 pathway [92]. Fucoidan treatment enhances the nuclear translocation of Nrf2 and binding to ARE, resulting in the upregulation of cryoprotective genes encoding antioxidant enzymes, as shown in Figure 1, including SOD, GSH, and CAT [92]. Ryu and Chung reported that fucoidan effectively attenuated oxidative stress in HaCat cells by inducing the expression of enzymes such as HO-1 and SOD-1 through the Nrf2 pathway. The authors reported that fucoidan activated the Nrf2 pathway by reducing the cytoplasmic stability of Keap1 [29]. Fucoidan also inhibited the Keap1-independent degradation of Nrf2 via the glycogen synthase kinase-3β (GSK3β) axis by increasing the phosphorylation of GSK-3β. Additionally, fucoidan treatment increased the expression of Nrf2 and HO-1, which together led to a reduction in the level of malondialdehyde (MDA) and reactive oxygen species induced by LPSs in acute lung injury [93]. Zhang et al. also reported that the antiaging activity of fucoidan in *Drosophila melanogaster* was mediated through the Nrf2 signaling pathway. The authors indicated that the administration of fucoidan induced the production of antioxidant enzymes, including SOD, CAT, and GSH-Px. This effect was linked to the upregulation of the expression of Nrf2 coupled with a downregulation of Keap1 in flies [94].

In wound healing, angiogenesis is a key step and is found to be regulated by the Nrf2/HIF-1α pathway. In this regard, fucoidan facilitated angiogenesis during wound healing by activating the AKT/Nrf2/HIF-1α pathway and the expression of downstream effectors, endothelial nitric oxide synthase (eNOS), and vascular endothelial growth factor (VEGF) [95]. Yu et al. showed that treatment with fucoidan induced the activity of Nrf2 while reducing the cytosolic expression of Keap1, leading to an increased expression of HO-1 in advanced glycation product (AGE)-stimulated rats [96]. Elsewhere, fucoidan inhibited the ferroptosis of hepatocytes partly through the upregulation of the p62/Nrf2 axis [97]. Additionally, fucoidan exhibited protective activity against H_2_O_2_-induced oxidative damage by increasing the translocation of Nrf2 from the cytosol into the nucleus. Subsequently, the mRNA levels of the downstream Nrf2-target genes, including NADH quinone dehydrogenase 1 (NQO1), SOD1, and GSH-Px, were significantly upregulated upon fucoidan treatment [98]. In diabetes, fucoidan has been shown to upregulate the Nrf2 pathway together with its respective downstream targets, thus delaying the pathological damage to the kidneys [99].

#### 3.1.6. Vascular Endothelial Growth Factor (VEGF)

VEGF is a diffusible and endothelial-specific mitogen produced by many cells, including macrophages, platelets, keratinocytes, renal mesangial cells, and tumor cells. It is crucial in the vascular system for stimulating angiogenesis and vascular hyperpermeability. It also plays important roles in bone formation, hematopoiesis, wound healing, etc. [100]. The activation and expression of VEGF are mainly regulated by hypoxia, i.e., mediated by the hypoxia-inducible factor and other factors such as epidermal growth factors and platelet-derived growth factors (PDGFs) [101,102]. The binding of VEGFs to their respective receptors promotes their interaction with proteins such as neuropilins, integrins, cadherins, and heparan sulfate proteoglycans [103]. These interactions have been implicated in the pathogenesis of diseases, including cancer, atherosclerosis, stroke, and cardiovascular disease, among others [100].

Fucoidan reportedly reduces the expression and production of VEGF at the onset of diseases. In this regard, Dithmer et al. reported that fucoidan significantly reduced the expression of VEGF in retinal pigment epithelium (RPE) cells and thus could be useful in the management of age-related macular degeneration [104]. Fucoidan also decreased angiogenesis through the downregulation of VEGF and stomal-derived factor-1 (SDF-1) [105]. Neuropilins (NRP)-1 and 2 are cell surface receptors that can transduce VEGF signals via VEGF receptor (VEGFR) 2 [106]. In this regard, fucoidan treatment has been reported to reduce the surface expression of NRP1 and NRP2 as well as VEGFR-1 and VEGFR-2 in primary human umbilical vein endothelial cells. The authors further demonstrated that fucoidan can suppress VEGF-induce angiogenesis and neovascularization in mice [106]. Also, the binding of fucoidan to VEGF_165_ competitively inhibited the interaction between VEGF and its receptor, VEGFR2. Moreover, fucoidan downregulated the level of VEGF secretion in ARPE19 cells [107]. In lung cancer cells, fucoidan inhibited tumor angiogenesis through the disruption of the VEGF–VEGFR2 interaction. This effect allowed for the blocking of signaling pathways, including VEGFR2 and ERK. The binding affinity of fucoidan to VEGFR2 was higher than that of VEGF, thus increasing stearic hindrance and preventing the further binding of VEGF after fucoidan was already bound [108].

Also, fucoidan has been found to exhibit antiangiogenetic and antitumor properties through the interaction of sulfate groups with VEGF. Negatively charged groups on fucoidan interacted with VEGF_165_ and blocked its recognition and binding to the receptor. Additionally, the proliferation and cell migration of human umbilical vein endothelial cells (HUVECs) were inhibited via the suppression of the phosphorylation of VEGFR2, which halted signal transduction [109]. Abdollah et al. reported that the attenuation effect of fucoidan against Avastin in HCC cells was stimulated by the inhibition of VEGF expression and secretion as well as the modulation of other signaling pathways, including PI3K/AKT/mTOR and the RAS/RAF/MAPK [110]. Elsewhere, the antiangiogenic effect of fucoidan in prostate cancer cells was mediated via the inhibition of the VEGF coupled with a reduced phosphorylation of the JAK-STAT3 pathway [111].

Conversely, fucoidan has been reported to promote the binding of VEGF_165_ to VEGFR-2 and NRP1 on endothelial cells, which could help in stimulating therapeutic revascularization [112].

#### 3.1.7. Tumor Necrosis Factor α (TNF-α) Pathway

TNF-α is a cytokine with pleiotropic effects and has been identified as the main regulator of inflammatory responses. TNF-α is known to be involved in both physiological and pathological processes [113]. TNF-α mediates responses to relevant stimuli by binding to tumor necrosis factor receptors (TNFR-1 and TNFR-2) and triggering cellular processes such as cell apoptosis and proliferation [114]. Upon the activation of TNF-α, two transcription factors, NF-κB and activating protein-1 (AP-1), are also stimulated. As such, the activation of TNF-α has been linked with the genesis of certain diseases, including inflammation, diabetes, obesity, cancer, and others, mainly via TNFR-1 [115,116].

Fucoidan has been involved in elucidating anti-inflammatory and anticancer activity through the downregulation of the TNF-α pathway. The application of fucoidan suppressed the phagocytic ability of porcine peripheral blood polymorphonuclear cells (PBMCs) by inhibiting the protein and mRNA expression of TNF-α in LPS-induced PBMCs [117]. Interestingly, the authors observed that although fucoidan treatment significantly suppressed the excessive production of TNF-α in LPS-induced PBMCs, the treatment of uninduced PBMCs with fucoidan also resulted in an increase in TNF-α production, albeit to a far lesser extent. Do et al. reported that fucoidan exerted anti-inflammatory activity by decreasing the production of nitric oxide through downregulating the expression of iNOS and AP-1 in TNF-α-stimulated cells. In addition, fucoidan treatment inhibited the TNF-α-induced activation of other pathways, including the p38 MAPK, JAK/STAT, and IRF-1 signaling pathways [118]. Similarly, fucoidan inhibited mRNA expression and the TNF-α-mediated activation of pathways such as NF-κB and MAPKs in human RPE cells [30]. Elsewhere, in hypoxia-induced lung injury, treatment with fucoidan reduced the production of cytokines, including TNF-α, IL-1, and IL-6. Subsequently, the decreased production of these cytokines inhibited the phosphorylation and expression of the ERK1/2 signaling pathway [119]. Fucoidan also exhibited anticancer activity against hepatocellular cells by reducing oxidative stress through the concomitant suppression of TNF-α and NF-κB [120].

In contrast, Jeong et al. reported that fucoidan exhibited a cryoprotective activity in dendritic cells by upregulating the production of TNF-α [121]. Furthermore, fucoidan inhibited the growth of A549 lung adenocarcinoma cells by increasing the secretion of TNF-α levels and other cytokines from the peritoneal macrophages into the serum, thus increasing the immune response [122].

### 3.2. Interaction with Receptors

#### 3.2.1. Toll-Like Receptors (TLRs)

TLRs are a class of PPRs commonly located in cell membranes, endosomes, and/or on different immune cells, including dendritic cells, macrophages, etc. They are involved in mediating inflammatory pathways and play a major role in the innate immune system [123]. They are made up of 10 different members, which assist in the recognition of specific microbial components, i.e., pathogen-associated molecular patterns (PAMPS), leading to the activation of innate immunity [124]. Upon recognition of PAMPs, a TLR initiates the transduction pathway, leading to the activation of NF-κB, IRFs, or MAP kinases, and in turn regulating the expression of cytokines, chemokines, and type I interferons (IFNs), which are mainly involved in protecting the host from microbial infection [125]. TLRs have been found to be involved in the pathogenesis of certain diseases, such as rheumatoid arthritis, tuberculosis, malaria, myocarditis, hepatitis, kidney failure, diabetes, and others [126,127,128]. As such, they have become one of the main targets for drugs and other bioactive compounds, including fucoidan, for the treatment of diseases.

Fucoidan has been found to exhibit immunomodulatory and anti-inflammatory activities through direct interaction with TLRs, especially TLR2 and TLR4. This interaction is facilitated through electrostatic forces acting between the negatively charged groups in fucoidan and the positively charged groups in TLRs [6]. Makarenkova et al. showed that fucoidan induced defense against pathogenic microorganisms through interactions with TLR2 and TLR4, which in turn activated the NF-κB pathway. The authors further revealed that the subsequent activation of NF-κB induced the expression of proinflammatory cytokine genes and interferon-inducible genes, leading to the assembly of immunocompetent cells as well as T cells in response to foreign materials [129]. Additionally, fucoidan induced macrophage activation through the stimulation of TLR2 and TLR4 [130]. Also, in lung cancer cells, it has been demonstrated that TLR4 knockdown inhibits fucoidan-induced apoptosis [131]. This corroborates the interaction of fucoidan with TLRs such as TLR4.

Interestingly, fucoidan has also been reported to downregulate the mRNA expression of TLR2 and TLR4 in activated macrophages after 6 h of incubation, thus inhibiting LPS-induced inflammation [27]. Similarly, Wang et al. reported that fucoidan reduced inflammation induced by LPSs by suppressing the TLR2- and TLR4-mediated activation of the NF-κB pathway [132]. Fucoidan also downregulated TLR4 expression in a diabetes mouse model, which culminated in a reduced inflammatory response in the pancreas, thus preventing further damage to pancreatic cells [133]. In obese mice, fucoidan prevented gut dysbacteriosis and insulin resistance by suppressing the TLR4 pathway and its downstream signaling pathways [134]. Also, the neuroprotective activity of fucoidan in alcohol withdrawal mice was reported to be linked with TLRs. Fucoidan was reported to suppress inflammation in the brains of mice by decreasing the expression of TLR4 and MyD88 as well as downregulating the phosphorylation of NF-κB p65 [135].

#### 3.2.2. Epidermal Growth Factor Receptor (EGFR)

Epidermal growth factor (EGF) is a polypeptide responsible for stimulating cell growth and differentiation. The activity of EGF is mediated by its receptor, EGFR, also known as erythroblastic leukemia viral oncogene homolog 1/human epidermal growth factor receptor 1 (ErbB1/HER1). This receptor is a tyrosine kinase and is involved in the development of many tumors such as in lung cancer, metastatic colorectal cancer, pancreatic cancer, breast cancer, and others [136]. In humans, HER2 has been studied and associated with breast cancer, thus becoming a known target for therapy. HER3, on the other hand, has been found to be an activator of other EGFRs. In addition, HER4 is associated with mutagenesis and differentiation [137]. The binding of EGFRs to ligands such as EGF, TNF-α, and Grb-2 results in the activation of other signaling pathways such as Ras, MAPK, ERK, and PI3K/AKT [138,139]. Also, the EGFR signaling pathways also lead to the progression of cells from the G1 phase to the S phase during the cell cycle upon activation with EGF [136]. Moreover, the EGF activation of EGFR facilitates cell proliferation [140,141]. Furthermore, mechanisms like mutation, receptor overexpression, and ligand-independent activation tend to activate EGFRs and promote tumor development. Thus, targeting these receptors, their downstream pathways, and ligands is important for preventing cancer and other diseases [142].

The existing literature showed that fucoidan exhibits some of its biological activity by regulating EGFR both in in vitro and in vivo studies. Oh et al. showed that fucoidan, together with the anticancer drug lapatinib (an inhibitor of tyrosine kinase), synergistically inhibited tumor development in EGFR/ERBB2-amplified cancer cell lines [143]. A similar observation was reported by Thakur et al., where a combination therapy involving fucoidan and lapatinib was effective in inhibiting melanoma growth. The authors revealed that the activity was linked with the blocking of ERBB3, either by a specific shRNA or a selective ERBB3 neutralizing antibody [144]. Furthermore, fucoidan exhibited anti-influenza activity by suppressing the activation of EGFR and its downstream pathways, NF-κB and AKT. In addition, fucoidan inhibited the internalization of EGFR in influenza A virus-infected cells, thus preventing sequences of endocytosis in cells [145]. Others have reported the effects of fucoidan on the sensitivity of sorafenib activity [146]. It was found that fucoidan inhibited cell migration in HepG2-SR. Subsequently, a combined treatment (fucoidan + sorafenib) blocked EGFR and its nuclear distribution into lipid rafts, as well as suppressing downstream transcription. Thus, fucoidan enhanced the sensitivity of sorafenib and its antitumor activity [146]. Lee et al. also demonstrated that the chemopreventive activity of fucoidan was facilitated by the inhibition of the EGF-induced phosphorylation of EGFR, which subsequently downregulated the phosphorylation and transactivation of the ERK and JNK signaling pathways in mouse epidermal cells and inhibited EGF-induced cell transformation [147]. Table 1 summarizes the potential targets and associated diseases treated with fucoidans based on previous in vivo studies performed to investigate their mechanisms of biological activities.

## 4. In Silico Studies

Molecular docking is a known in silico structure-based technique used in drug development and in-depth analyses of the interaction between ligands and proteins [177,178]. In this review, critical proteins in different diseases were chosen to understand how prospective fucoidan interacts with some proteins for therapeutic applications.

### 4.1. Results and Discussion

**Figure 2:**
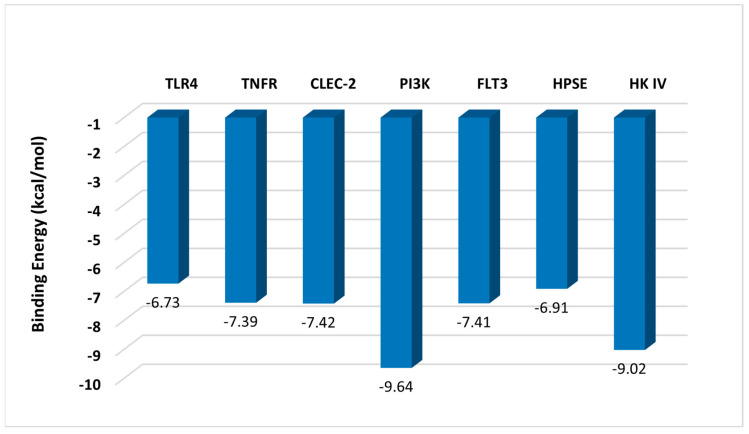
Binding free energy of fucoidan–protein complexes.

The binding energy of fucoidan’s interactions with different proteins is shown in Figure 2. Fucoidan has a high affinity to PI3K (−9.64 kcal/mol) and Hexokinase IV (−9.02 kcal/mol). The remaining proteins also had a high affinity with different types of interactions. Overall, the sulphate groups in fucoidan played a significant role in its interactions with proteins. The negatively charged sulphate groups interact electrostatically with positively charged regions of proteins, i.e., arginine residues. In addition, the hydroxyl groups on tyrosine residues participate in hydrogen bonding with the hydrogen bond acceptors on the sulphate group in fucoidan. These hydrogen bonds may influence the stability of the fucoidan–protein complex. Below are some detailed potential mechanisms of how fucoidan interacts with each targeted protein.

#### 4.1.1. Predicted Interaction of Fucoidan with Receptors

##### Inhibition Effect of TLR4 and TNFR

**Figure 3:**
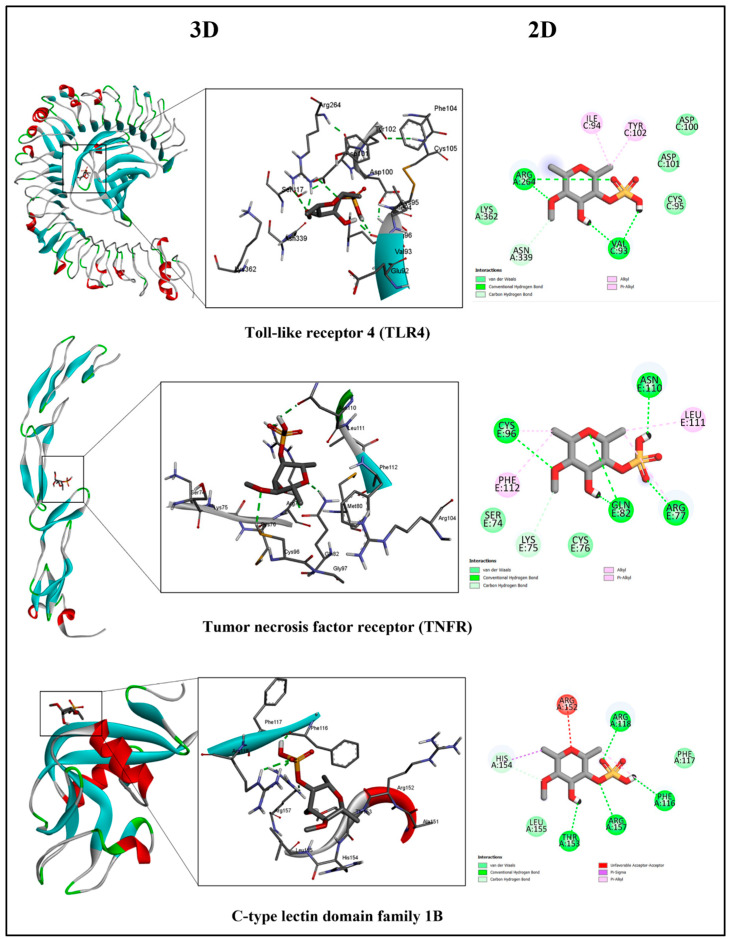
Molecular docking analysis of fucoidan–receptor complexes. The interaction of fucoidan and proteins is represented as 3D ribbon structures with a magnification of the binding sites of its interaction in 3D (green dashes represent hydrogen bonds) and 2D.

It has been demonstrated that the nucleotide-binding domain, the leucine-rich-containing family, and pyrin domain-containing-3 (NLRP3) inflammasome suppression were effective in managing several inflammatory diseases. The key molecular mechanism of NLRP3 inflammasome activation was attributed to the NF-κB signaling pathway. Therefore, targeting immune receptors such as TLR4 and TNF to inhibit NLRP3 is an effective method to enhance NLRP3 inflammasome activation [179,180]. Pharmaceuticals that modulate TLR activation are very interesting due to their therapeutic potential. Several studies have reported the potential application of TLR4 antagonists in treating inflammatory disorders [181,182,183]. TLRs typically act as heterodimers and recognize numerous ligands with distinctive pathogen-associated molecular patterns (PAMPs). The co-receptor for TLR4, myeloid differentiation protein 2 (MD-2), is critical in the signaling and ligand selectivity of TLR4. After the ligand binds to the extracellular domains, the TLR4–MD-2 complex is rearranged, thus activating downstream inflammatory cascades [184]. During endotoxic signalling, the Phe126 residue in the hydrophobic pocket of MD-2 represents the “molecular switch”. This residue is linked to TLR4 activation and allosterically triggers a conformational shift in a ligand-dependent way [185,186]. Others have reported that during activation of TLR4 via endotoxin, Phe121, Phe126 and Tyr131, played a crucial role in human MD-2 [187]. The docking results showed that fucoidan did not interact with these residues (Figure 3). This implies that fucoidan antagonists TLR4MD-2 may inhibit the activation of the dimerization complex, thus suppressing NLRP3 activation.

In addition, the molecular docking results of fucoidan against the TNFR (Figure 3) show the interacting residues that formed H-bonds with Arg77 and Asn110 and the hydrophobic interactions with Ser74 and Lys75. These residues participated in the interaction of TNF-α and Ternatin with TNFR [188] at the binding site, which suggests the suppressing potential of the NLRP3 inflammasome supported an in vivo relief of gastric ulcers [22].

##### Activation Effect of CLEC-2

Rhodocytin is an endogenous ligand that activates the C-type lectin-like protein (CLEC-2) through interaction at the 132, 150, 168, 171, 184, 187, 188, 190, 192, 200, and 211 residues [183]. On the other hand, podoplanin, an exogenous ligand, binds to the other residues of rhodocytin and forms *O*-glycosylation with the CLEC-2 protein [189]. Fucoidan has a high affinity to bind to CLEC-2 at −7.42 kcal/mol, with residues similar to those in its interaction with podoplanin. In addition, it is stabilized by four hydrogen bonds, where three are formed via a sulfate group (Arg118, Arg157, and phe118), and the last via an oxygen atom with Thr153 (Figure 3). The NetOGlyc-4.0 server predicted that the protein sequence may contain *O*-glycosylation sites, and it projected that Thr153 on CLEC-2 would be one of the sites that *O*-glycosylated upon attachment to sugar. The accumulated evidence demonstrated that fucoidan is an agonist against CLEC-2 and a platelet activator [83,190,191]. It is possible that fucoidan binding stimulates or activates glycosylation at Thr153, which may influence protein structure, function, and localization, despite the lack of prior research.

#### 4.1.2. Interaction of Fucoidan with Enzymes

##### Inhibition Effect of PI3K and FLT3

Regarding the interaction of fucoidan with PI3K, a lipid kinase is essential for several biological processes, including intracellular signaling, cell growth, survival, and proliferation via the PI3K/AKT/mTOR signaling pathway. PI3K gamma (PI3Kγ) is an enzyme that belongs to this family of PI3K and is considered as a promising therapeutic agent for cancer, inflammation, and autoimmune diseases. PI3Kγ has two subunits (catalytic and regulatory) and N- and C-terminal lobes, which form a deep edge with the adenosine triphosphate (ATP)-binding pocket [192]. The docking analysis showed that fucoidan bonded to the ATP binding site, forming three hydrogen bonds with residues (Tyr867, Glu880, and Val882), and hydrophobically interacted with Met804, Ile831, Ile879, Ile963, and Asp964 residues (Figure 3), similar to residue interaction with other inhibitors [193,194,195]. Considering the fact that fucoidan had a high binding affinity with PI3K and targeted the ATP-binding pocket, this may influence the enzyme’s binding properties and selectivity; thus, it is imperative to decipher the probability of kinase inhibition.

**Figure 4:**
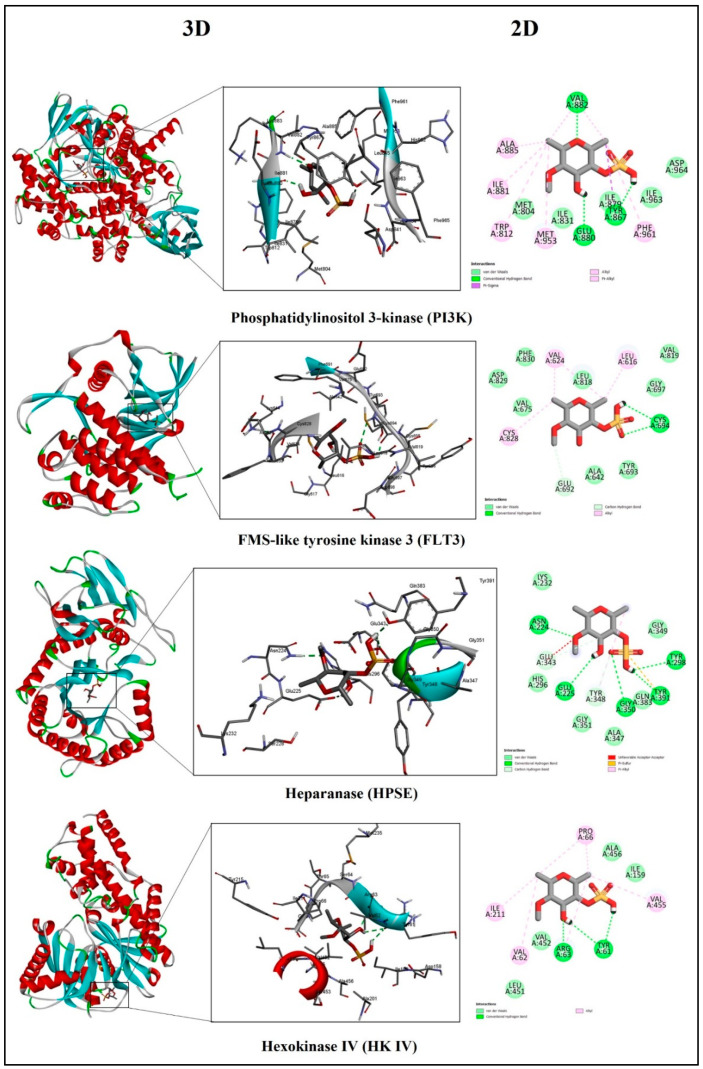
Molecular docking analysis of fucoidan–enzyme complexes. The interaction of fucoidan and proteins is represented as 3D ribbon structures with a magnification of the binding sites of its interaction in 3D (green dashes represent hydrogen bonds) and 2D.

In normal physiology, the FLT3 receptor dimers, when bound to their ligand, activate conformation, which initiates downstream signaling. Generally, FLT3 inhibitors are classified based on their interaction site. Type I binds to the active conformation of the ATP-binding pocket, inhibiting it competitively, whereas Type II inhibitors interact with the hydrophobic region next to the ATP-binding site [196]. The lengthy, flexible peptide region which constitutes FLT3′s activation loop is found at both the N- and C-terminal ends and contains the highly conserved DFG (Asp829-Phe830-Gly831) motif. Asp829 acts as the catalytic base in the transfer of a phosphate group and is invariant in kinases [197]. Figure 4 shows fucoidan’s interaction with an active conformation of FLT3, which is stabilized by −7.41 kcal/mol through two hydrogen bonds with Cys694, as well as one carbon–hydrogen bond with Glu692, and other non-covalent interactions, specifically with Asp829 and Phe830 via VDW. These similar residues on the kinase regions interacted with Gilteritinib, an FDA-approved inhibitor and a Type I FLT3 inhibitor [198,199] and enhanced the stability of the inhibitor–protein complex. As a result, fucoidan may inhibit kinase activation even when it does not interact with the gatekeeper residue (F691). Therefore, fucoidan inhibited FLT3 from phosphorylating tyrosine residues and thus initiated subsequent signaling.

Heparan sulphate chains in extracellular matrixes and cellular membranes are broken down by the enzyme heparanase (HPSE), which influences cell adhesion, migration, invasion, and tissue integrity. As a result, HPSE activity is dysregulated, making it a desirable target for anti-inflammatory, antiangiogenic, and antimetastatic drugs [200]. Sulphated polysaccharides and oligosaccharides have been suggested as potential HPSE inhibitors, while the residues Glu343 and Glu225 have previously been identified as HPSE proton donors and nucleophiles [201,202]. In the docking interaction of the fucoidan–HPSE enzyme (Figure 4), the hydroxyl group of Glu225 formed hydrogen bonds with the sulphate group, whereas the carboxylate group on Glu343 interacted via van der Waals forces. Due to this electrostatic interaction with these catalytic nucleophiles, fucoidan’s sulphate group may disrupt catalytic function and decrease enzymatic activity. Furthermore, these non-covalent (hydrogen bond and hydrophobic) interactions enhanced the stabilization of fucoidan in HPSE, and covalent interactions via carbon–hydrogen binding, which were formed with Tyr348, may be sufficient to bind to the enzyme with fucoidan and exert pharmacological activity.

##### Stimulation Effect of HK IV

It is important to note that the challenge in the development of antidiabetic drugs is the activation of hexokinase [203], which regulates glucose homeostasis. Hexokinase IV (HK IV) has two sites: an active site, which binds with its substrate (i.e., glucose), and an allosteric site (for the activator). Therefore, fucoidan in the allosteric site of HK IV was docked to predict the probability of its activation. The docking results (Figure 4) showed that fucoidan binds to the agonist-binding residues of HK IV [204,205] to form three hydrogen bonds (two with Try61 and one with Arg63). Alkyl and VDW interactions may enhance the binding affinity and stability of the HK IV–fucoidan complex. In addition, fucoidan binding to the allosteric site may stimulate HK IV activity and thus improve glucose metabolism. It is worth mentioning that the combined effects of low-molecular-weight fucoidan (LMWF) and fucoxanthin dramatically enhanced the overexpression of insulin receptor substrate-1 (IRS-1) and glucose transporter type 4 (GLUT4) in a mouse model of type II diabetes (T2D) [206]. An integrated experimental investigation is warranted to ensure accuracy and decipher the relevance of the pharmacological effect of fucoidan on interactions between ligands and proteins via molecular docking.

### 4.2. Methods

#### 4.2.1. Preparation of Ligand

The 3D structure of a monomer fucoidan unit (CID: 129532628) was retrieved from the PubChem database (accessed on 23 July 2023) in SDF format and converted to pdbqt format using Open Babel-3.1.1 [207]. The SwissTargetPrediction and Super-PRED (accessed on 27 July 2023) webservers predicted the biological targets of *Homo sapiens* using 3D-structured fucoidan. Based on the prediction results coupled with the existing literature, the main receptors involved in single transduction and enzymes were selected.

#### 4.2.2. Preparation of Proteins

The 3D X-ray structures of proteins, including TLR4, TNFR, CLEC-2, PIK3, FLT3, HPSE, and HK IV, were obtained from the Protein Data Bank (PDB ID: 3FXI [181], 1EXT [208], 2C6U [209], 3DBS [210], 6JQR [196], 5E9C [200], and 3F9M [211], respectively, accessed on October 1, 2023). These proteins were obtained when water and co-crystallization molecules were removed before hydrogen bonds were added to minimize energy use using Chimera 1.16 software. The missing residues in 6JQR were built using homology modelling via the SWISS-MODEL webserver [212]-based sequencing protein in UniPort (P36888 FLT3_HUMAN, (accessed on 27 September 2023)).

#### 4.2.3. Molecular Docking

The binding free energy (kcal/mol) of protein–fucoidan complexes was calculated with AutoDockTool-1.5.6. The complex was chosen based on the lowest docking energy score and visualized with Discovery Studio V21.1.0.

## 5. Toxicity Studies

As presented in the manuscript and many other literature sources, fucoidan is acknowledged to exhibit numerous biological activities. However, it is imperative to ascertain its safety to promote its application in the pharmaceutical, food, and cosmetic industries. Generally, fucoidan is widely perceived as non-toxic, biodegradable, and biocompatible [23]. These assertions are corroborated by several scientific studies. For instance, Lim et al. reported no mortality or adverse reactions in Sprague-Dawley rats after the administration of fucoidan at a dose of 2000 mg/kg body weight for 14 days. Other studies likewise reported that the administration of fucoidan at a dose of 40 mg/kg to mice for 14 days did not induce toxicity in the liver or kidneys [173]. Elsewhere, the oral administration of fucoidan at a dose of 1350 mg/kg for 4 weeks in Sprague-Dawley rats did not induce toxic effects and was considered safe for further utilization. Furthermore, a repeated-dose oral toxicity assessment in rats showed that the administration of fucoidan up to 2000 mg/kg over 28 days showed no toxicological effects in terms of hematological and biochemical parameters as well as organ damage. Following an in vivo micronucleus assay, the authors also observed no mutagenic potential of fucoidan at a dose of 2000 mg/kg in mice [213]. Kim et al. likewise observed that an oral gavage of fucoidan (2000 mg/kg/day) did not induce cytotoxicity or genotoxicity in mice [53]. Also, acute and subacute toxicity studies involving the oral administration of 2000 mg/kg of fucoidan revealed no adverse reactions, mortality, or alterations in physiological parameters in mice over a 28-day period [214]. The safety of fucoidan is further revealed in another study involving the oral administration of fucoidan (up to 1000 mg/kg) for 14 days in Sprague-Dawley rats [215].

Additionally, an in vitro cytotoxicity MTT assay showed no adverse effects of fucoidan (6.25–50 mg/mL) on normal human cell lines [216]. Furthermore, the treatment of HEK293 eukaryotic cells with fucoidan effectively regulated molecular targets such as TLRs, NF-κB, and β-galactosidase without any noticeable adverse effects [129]. In the context of gastric cancer treatment, reports have frequently documented various adverse reactions. However, a treatment with fucoidan at a concentration of 200 µg/mL did not result in toxicity to gastric mucosal epithelial cells after 3 days [83]. Also, Hwang et al. employed methods such as a bacterial reverse mutation assay, a chromosome aberration assay, and a micronucleus assay to determine the toxicological effect of LMWF in mice. Notably, LMWF at a concentration of 5000 μg/mL exhibited no mutagenicity [213]. Additionally, high-molecular-weight fucoidan (HMWF) exhibited no genotoxic effect in a reverse mutation assay, micronucleus assays, and a chromosomal aberration assay [53,217]. Additional results in vitro also revealed no toxicity to rabbit articular chondrocytes [215].

In contrast to the above observations regarding the safety of fucoidan, a study by Chung et al. revealed that the administration of fucoidan (2000 mg/kg) altered the activity of the liver enzyme alanine transaminase as well as the metabolism of lipoprotein in Sprague-Dawley rats [218]. Additionally, although the administration of 900 and 2500 mg/kg of fucoidan in rats showed no toxic effects, the authors indicated the possibility of it causing renal problems at these doses [219]. The administration of fucoidan (25 mg/kg) in C57BL/6 mice exerted a toxic effect, leading to the death of 10 mice in a period of 20 days. It is worth noting that the repeated administration of 10 mg/kg of fucoidan on days 3, 8, and 12 in the same experiment revealed no adverse effect in mice [220]. In vitro studies also revealed that fucoidan exhibited mild cytotoxicity at concentrations <200 µg/mL, with significant cytotoxicity occurring at ≥300 μg/mL in rat intestinal crypt epithelial cells (IEC-6). These effects were associated with the presence of polyphenols in the fucoidan extract [221].

In addition to animal and in vitro studies, several reports have highlighted the safety of fucoidan in human trials. Clinical studies involving the administration of 99mTechnetium-labeled (99MTC) fucoidan as a diagnostic agent for P-selectin imaging in 10 patients revealed no adverse reactions up to 24 h after administration [222]. Also, the oral administration of fucoidan (4000 mg/day) to 20 Japanese patients aged between 18 and 76 revealed no toxicity on the liver, kidney, or other organs after a duration of 4 weeks [223]. The administration of fucoidan (1000 mg; 500 mg in the morning and in the evening) to 10 Australian patients showed no adverse effects or signs of toxicity. After 3 weeks of administration, the participants reported no discomfort during subsequent follow-ups [224]. Similarly, the oral administration of approximately 4 g of fucoidan to 20 patients for two weeks revealed no toxicity and, as such, was recommended for consideration in the treatment of atherosclerosis [225]. Other studies revealed that the administration of fucoidan (100 and 1000 mg) supplemented with vitamin B6, zinc, and manganese for 12 weeks was generally considered safe. However, the authors reported incidents of adverse effects, including reports of hypertension (2 participants), chest infection (one participant), hyperacidity (one participant), and a root canal (one participant) during the 12-week administration. These events are thought to be associated with the patients’ histories rather than the administration of fucoidan [226]. In a study involving the administration of a 6 g dose of fucoidan to 13 patients with HTLV-1-associated myelopathy/tropical spastic paraparesis for 13 months, the onset of diarrhea was reported in four patients during the intervention period, with no adverse events recorded for the other nine patients [227]. A pre-clinical study likewise found fucoidan (0.2 mg/mL) from *F. vesiculosus* and *U. pinnatifida* used in cancer treatment to be safe. However, when combined with chemotherapy, certain toxicities were induced in human cancer mouse models [228].

## 6. Conclusions

Fucoidan exerts highly promising bioactivities targeting specific receptors and enzymes. These molecular targets are associated with a wide spectrum of diseases, ranging from simple inflammation to cancers. The anionic characters and molecular weight of fucoidan seem to contribute potentially to most of its activity, either through activation or inhibition effects. Examples of these targets include lipid kinase, heparanase, and hexokinase, in addition to TNF-α, TGF-β, and VEGF. Moreover, toxicity studies have shown its safety over a wide range of doses. Despite accumulating evidence regarding the safety of fucoidan, further research is warranted, particularly in exploring the long-term toxicity of fucoidan. The current article may help explain the potential pharmacological activities of fucoidan performed in previous in vivo studies, where in silico studies consistently showed good docking scores. Furthermore, understanding its exact molecular mechanism may promote the semi-synthesis of novel fucoidan-based drug candidates to improve their efficacy in treating life-threatening diseases.

## Figures and Tables

**Table 1:** A summary of potential targets and associated diseases treated with fucoidans based on in vivo studies performed to investigate its mechanisms of biological activities.

Target	Associated Disease(s)	Experimental Model	Dose/Dosage of Fucoidan	Mechanism of Action	Biological Effect	Ref.
NF-κB	Chronic inflammation and cancer	Wistar rat	100–300 mg/kg/day	Suppresses IκB degradation ↑Expression of IκB	-Suppression of the inflammatory response and oxidative stress -↓COX-2 and iNOS -↓TNF-α, IL-1β, and IL6 -↑IL10	[148]
	Leukemia	HUT-102 cells	3 mg/mL for 72 h	↓Phosphorylation of IκBα	-Induction of apoptosis and cell cycle arrest -↓Survivin and cyclin D2 -↓cIAP-2 and c-myc	[149]
	Ophthalmic inflammation	ARPE-19 cells	1–50 µg/mL for 24 h	↓Phosphorylation of NF-κB	-Reduces inflammation and macular disorders -↓IL-6, IL-1ß, and IL-8 -↓TNF-α	[30]
	Chronic inflammation	THP-1 human monocytic cell	10–200 µg/mL for 24 h	↓Transcription of NF-κB	-Attenuation of pro-inflammatory cytokines in macrophages -↓COX-2 and iNOS -↓TNF-α, IL-1β, and IL6	[150]
	Inflammatory injuries	Male Swiss albino mice	50 and 100 mg/kg/day for 21 days	↓Translocation of NF-κB from cytoplasm to nucleus	-Alleviates hepatic, renal, and oxidative stress and inflammatory injuries -↓TNF-α, IL-1β, and IL6	[151]
	Diabetic neuropathy (DN)	Male GK and Wistar rats	10–1000 µg/mL for 24 h	↓Nuclear translocation of NF-κB-p65	-Reduces hyperglycemia and impedes development of DN -↓TGF-β1, and FN	[152]
	Abdominal aortic aneurysm	Angiotensin-II-induced mice	100 mg/kg/day for 28 days	↓Nuclear translocation of NF-κB-p65	-Attenuates elastin degradation and decreases macrophage infiltration -↓MMP-2 and MMP-9	[153]
MAPK	Cerebral Ischemia–Reperfusion Injury (IRI)	Male Sprague-Dawley (SD) rats	80 and 160 mg/kg/day for 7 days	↓Phosphorylation of ERK, JNK, and p38	-Elucidates a protective activity in cerebral IRI -↓p-p53 -↓Bax -↑Bcl2	[154]
	Renal Ischemia–Reperfusion Injury	Male C57BL/6J mice	100 mg/kg/day for 7 days	↓Phosphorylation of MAPK pathways	-Ameliorates acute renal IRI -↓Cytochrome c -↓p53 -↓Bax/Bcl2	[155]
	Bone development	Human alveolar bone marrow	0.1–10 µg/mL	↑Phosphorylation of ERK, JNK, and p38	-Promotes osteoblast differentiation -↑BMP2 -↑Smad 1/5/8	[156]
	Inflammation	RAW 264.7 macrophage cells	25 µg/mL for 24 h	↓Phosphorylation of ERK, JNK, and p38	-Reduces inflammation and cell death in cells -↓IL-6 -↓IL-1β -↓TNF-α	[33]
	Breast cancer	Female Spraque-Dawley rats	200 and 400 mg/kg/day for 16 weeks	↑Expression of ERK and p38 MAPK	-Modulates intestinal flora and inhibits tumor growth	[157]
	Cancer	Human cancer cell line (A549)	50–200 µg/mL for 24 h	↑Phosphorylation of ERK↓Phosphorylation of p38	-Impedes tumor growth in lung cells upon induction of apoptosis -↓Bcl2 -↑Bax	[158]
PI3K/AKT	Hypertension	Spraque-Dawley rats	20 and 100 mg/kg/day for 5 days	↑Phosphorylation of AKT and eNOS	-Reduces inflammation and oxidative stress and prevents hypertension -↑NO promotion in HUVECs	[159]
	Bladder cancer	Human bladder cancer cell	100 mg/kg/day	↓Expression of PI3K/AKT pathway	-Induces apoptosis in bladder cancer cells -↑Apoptosis and antitelomerase activity	[160]
	Colon cancer	HT-29 human colon adenocarcinoma cells	250 µg/mL for 24 h	↓Phosphorylation of PI3K/AKT	-Attenuates cell proliferation and induces apoptosis -↓IGF-IR	[161]
	Colon cancer	HT29 colon cancer cells	100 μg/mL for 24 h	↓Phosphorylation of PI3K/AKT	-Ameliorates growth of tumors and angiogenesis in cells -↓CDK2 and CDK4 levels	[72,73]
	Cancer	C57BL/6 mice and HUVECs	20–75 μg/mL daily for 7 days	↓Expression of PI3K/AKT↓Phosphorylation of mTOR	-Inhibits angiogenesis -↓Expression of HIF-1α and VEGF	[162]
TLR	Inflammation	RAW 264.7 cells	200 μg/mL for 48 h	↓Expression of TLR2 and TLR4	-Reduces inflammatory cytokines -↓MyD88	[163]
	Inflammation	RAW 264.7 cells	25–200 μg/mL for 24 h	↓mRNA expression of TLR2 and TLR4	-Decreases inflammatory mediators -↓JNK -↓ERK -↓p38 MAPK	[164]
	Airway inflammation	Bronchial epithelial cells and lung tissues	10 μg/mL for 24 h	↓Expression of TLR3	-Reduces viral infection and inflammations in the bronchioles -↓IL-6, TNF-α, IL-1α, and IL-1β	[165]
TGF-β	Kidney fibrosis	Renal tubular epithelial cell line	40–640 μg/mL for 72 h	↓Expression of TGF-β	-Ameliorates fibroid regeneration in renal tubular epithelial cells -↓Fibronectin and CTGF	[166]
	Kidney fibrosis	Renal proximal tubular cell line	40 μg/mL for 72 h	↓Expression of TGF-β	-Prevents progression of renal epithelial mesenchymal transition (EMT) -↓Fibronectin and alpha-smooth muscle actin	[167]
	Tubulointerstitial fibrosis	Chronic kidney disease mice	100 mg/kg/day	↓Expression of TGF-β	-Improves renal function and reduces tubulointerstitial fibrosis -↓CD44	[168]
	Pulmonary fibrosis	Male C57BL/6J mice	50–200 mg/kg/day for 16 days	↓Expression of TGF-β	-Attenuates inflammatory reaction and progression of EMT -↓Collagen 1 -↓PI3K/AKT	[169]
VEGF	Age-related macular degeneration	RPE cells	50 μg/mL for 6 h	↓Expression of VEGFR2	-Inhibits inflammation and offers protection against ocular disorders -↓ERK signaling pathway	[170]
	Breast cancer	Female Balb/c mice	10 mg/kg/day for 20 days	↓Expression of VEGR	-Suppresses angiogenesis and lung metastasis in breast cancer cells -↓Bcl-2 -↓ERK signaling pathway	[52]
	Diabetic retinopathy	Male C57BL/6 mice	50–200 mg/kg/day for 4 months	↓Secretion of VEGR	-Reduces hyperglycemia and attenuates neovascularization and retinopathy -↓Hypoxia-inducible factor-1α (HIF-1α)	[171]
EGF	Breast Cancer	Human TNBC cell lines	400 μg/mL/day	↓Expression of EGF	-Inhibits metastasis in breast cancer cells -↓IL-6 and PD-L1	[172]
Nrf2	Liver and kidney injury	Male ICR mice	20 and 40 mg/kg/day for 14 days	↑Expression of Nrf2 and HO-1	-Ameliorates liver and kidney injury and prevents oxidative stress -↓ALT, AST, CRE, and BUN -↓Activity of MDA -↓Production of IL-6, IL-1β, TNF-α -↑SOD, CAT, and GSH-Px	[173]
	Oxidative damage	Vero cells and H_2_O_2_-induced zebrafish	25, 50, and 100 μg/mL/day for 3 days	↑Expression of Nrf2 and HO-1	-Attenuated oxidative damage and suppressed heartbeat disorder. -↑SOD -↑CAT	[174]
	Diabetic cardiomyopathy (DCM)	Alloxan-induced DCM Wistar rats	150 mg/kg/day for 30 days	↑Translocation of Nrf2 from the cytoplasm into nucleus.	-Reduced oxidative stress in DCM. -↑SOD1, HO-1, NQO1, and CAT -↓MDA	[175]
	Ulcerative colitis (UC)	UC-induced Sprague Dawley rats	150 mg/kg/day for 2 weeks	↑Expression of Nrf2 and HO-1	-Ameliorated ulcerative colitis in rats. -↓MDA and peroxynitrite	[176]

↑ = increase, ↓ = decrease.

## Data Availability

Data are available from the corresponding author upon request.
